# Quality of Information in Gallstone Disease Videos on TikTok: Cross-sectional Study

**DOI:** 10.2196/39162

**Published:** 2023-02-08

**Authors:** Fei Sun, Shusen Zheng, Jian Wu

**Affiliations:** 1 Division of Hepatobiliary and Pancreatic Surgery, Department of Surgery The First Affiliated Hospital Zhejiang University School of Medicine Hangzhou China; 2 Key Laboratory of Combined Multiorgan Transplantation National Health Commission Hangzhou China; 3 Key Laboratory of Organ Transplantation Hangzhou China; 4 Zhejiang Provincial Research Center for Diagnosis and Treatment of Hepatobiliary Diseases Hangzhou China

**Keywords:** hepatobiliary, gallstone, gallbladder, TikTok, social media, video quality, DISCERN, Journal of American Medical Association, JAMA, Global Quality Score, GQS, content analysis, health information, online health information, digital health, disease knowledge, medical information, misinformation, infodemiology, patient education, dissemination, accuracy, credibility, credible, reliability, reliable, information quality

## Abstract

**Background:**

TikTok was an important channel for consumers to access and adopt health information. But the quality of health content in TikTok remains underinvestigated.

**Objective:**

Our study aimed to identify upload sources, contents, and feature information of gallstone disease videos on TikTok and further evaluated the factors related to video quality.

**Methods:**

We investigated the first 100 gallstone-related videos on TikTok and analyzed these videos’ upload sources, content, and characteristics. The quality of videos was evaluated using quantitative scoring tools such as DISCERN instrument, the Journal of American Medical Association (JAMA) benchmark criteria, and Global Quality Scores (GQS). Moreover, the correlation between video quality and video characteristics, including duration, likes, comments, and shares, was further investigated.

**Results:**

According to video sources, 81% of the videos were posted by doctors. Furthermore, disease knowledge was the most dominant video content, accounting for 56% of all the videos. The mean DISCERN, JAMA, and GQS scores of all 100 videos are 39.61 (SD 11.36), 2.00 (SD 0.40), and 2.76 (SD 0.95), respectively. According to DISCERN and GQS, gallstone-related videos’ quality score on TikTok is not high, mainly at fair (43/100, 43%,) and moderate (46/100, 46%). The total DISCERN scores of doctors were significantly higher than that of individuals and news agencies, surgery techniques were significantly higher than lifestyle and news, and disease knowledge was significantly higher than news, respectively. DISCERN scores and video duration were positively correlated. Negative correlations were found between DISCERN scores and likes and shares of videos. In GQS analysis, no significant differences were found between groups based on different sources or different contents. JAMA was excluded in the video quality and correlation analysis due to a lack of discrimination and inability to evaluate the video quality accurately.

**Conclusions:**

Although the videos of gallstones on TikTok are mainly provided by doctors and contain disease knowledge, they are of low quality. We found a positive correlation between video duration and video quality. High-quality videos received low attention, and popular videos were of low quality. Medical information on TikTok is currently not rigorous enough to guide patients to make accurate judgments. TikTok was not an appropriate source of knowledge to educate patients due to the low quality and reliability of the information.

## Introduction

Gallstone disease is a common disease worldwide. It occurs in 10%-15% of adults in the United States and Europe [1]. Most patients with gallstones are asymptomatic, often detected during physical examinations or other diseases, and do not require medical treatment. However, some patients with gallstones can be induced by acute or chronic inflammation of biliary colic, vomiting, diarrhea, and right upper abdominal tenderness, and serious cases can lead to cholangitis, biliary pancreatitis, gallbladder cancer, and other risks [1]. The standard treatment of symptomatic gallstone disease is laparoscopic cholecystectomy [2]. Currently, over 700,000 cholecystectomies are performed in the United States each year, with health care costs over US $6.5 billion [3]. Risk factors for gallbladder stones include the patient’s age, gender, race, family history, and metabolic diseases such as obesity, hypertension, and type 2 diabetes [4]. Changing the lifestyle of potential patients through health education can reduce the incidence of gallstones in a certain sense [5].

The unprecedented growth of web-based medical information has significantly changed the way people obtain health information. Nowadays, more and more patients seek information online before seeing a doctor [6]. With the increase in video content, videos have become one of the most critical ways for people to obtain medical information. However, the quality of web-based health-related videos is far from satisfactory. Over one-quarter of the most viewed YouTube videos on COVID-19 contained misleading information [7]. Furthermore, more than half of gallstone videos on YouTube were unreliable, with no correlation between video quality and the number of views or likes [8]. There is still much room for improvement in the quality of health-related videos.

Although health content has been widely studied on video sites such as YouTube, it remains underinvestigated in emerging short-video apps such as TikTok [9]. TikTok is available in over 150 countries, has over 1 billion users, and has been downloaded over 200 million times in the United States alone [10]. On TikTok, users can create their own videos by lip-synching or dancing along with popular songs. TikTok is not just about entertainment; it also contains many health care–related content [9,11]. A recent study suggests that TikTok could be an important channel for consumers to access and adopt health information [12]. TikTok has huge potential to better serve public health communication [13]. Researchers have explored the video quality of COVID-19, diabetes, and chronic obstructive pulmonary disease (COPD) on TikTok [14-16]. However, gallstone contents on TikTok have not been studied. Therefore, we investigated the first 100 gallstone-related videos on TikTok. This study aimed to identify upload sources, contents, and feature information of these videos and evaluate the information quality of gallstone videos on TikTok by using quantitative scoring tools such as DISCERN, Journal of American Medical Association (JAMA) benchmark criteria, and Global Quality Scores (GQS). We further investigated the correlation between video likes, comments, and shares, on the one hand, and video information quality, on the other.

## Methods

### Ethical Considerations

No clinical data, human specimens, or laboratory animals were involved in this study. All information used in this study was obtained from publicly released TikTok videos, and none of the data involved personal privacy. In addition, the study did not involve any interaction with users; therefore, no ethics review was required.

### Data Collection

In this cross-sectional study, the Chinese version of TikTok was searched on July 18, 2020, using the term gallstone in Chinese. Videos were sorted by comprehensive ranking with release time set to unlimited, which is the TikTok default. Non-Chinese videos were excluded until the top 100 videos in Chinese were displayed. We limited our analysis to the top 100 videos because multiple studies have confirmed that videos beyond the top 100 had no significant impact on the analysis [17,18]. For each TikTok video concerning the gallstone, the following characteristics were recorded and analyzed: title, number of likes, number of comments, number of shares, tags, upload date, download date, days since upload, video duration, content, and video source (uploader). Research has found that background music does not significantly increase the popularity of videos [17]. Therefore, this study mainly focuses on the content of the video; the quality of the video image and the music contained in the video are not within the scope of this study.

### Classification of Videos

Video sources were categorized as follows: (1) doctors, (2) individuals (ie, nonmedical professionals), (3) news agencies (ie, network media, newspaper, TV station, and radio station), and (4) organizations (ie, hospitals, health authorities, research groups, universities, or colleges). The content was categorized as follows: (1) surgery technique, (2) disease knowledge, (3) lifestyle, and (4) news. This classification allows us to group videos with the same content as much as possible, while distinguishing videos with different content.

### Quality Assessment

The quality of the information in videos was assessed using the DISCERN instrument, the Journal of American Medical Association (JAMA) benchmark criteria, and Global Quality Scores (GQS). The DISCERN instrument was developed for judging the quality of health information on treatment choices [19]. It includes 16 questions categorized into 3 sections on a 5-point scale (Multimedia Appendix 1). Section 1 (questions 1 to 8) assesses the reliability of an article, section 2 (questions 9 to 15) focuses on the quality of treatment information, and section 3 (question 16) evaluates the overall quality [20]. The overall DISCERN scores ranged from 16 to 80 and were categorized as very poor (16-26), poor (27-38), fair (39-50), good (51-62), and excellent (63-80) [21,22]. The JAMA benchmark criteria evaluated video source reliability ranges from 0 to 4 [23]. They consist of 4 individual criteria and are assigned 1 point for each (Table 1). A score of 4 indicates higher quality, while a score of 0 indicates poor quality. The GQS assesses educational value through 5 criteria (Table 2) [24]. The GQS scores range from 1 to 5. The maximum score of 5 indicates high quality.

All authors were senior surgeons engaged in hepatobiliary and pancreatic surgery for years and were knowledgeable in diagnosing and treating gallstone diseases. On the process for screening and rating, authors FS and JW used the DISCERN instrument, JAMA benchmark criteria, and GQS to evaluate the videos contemporaneously. The scores were determined through discussions. An arbitrator (SZ) resolved inconsistent scores between viewers and gave the final scores. Subsequently, all authors agreed on all the ratings.

**Table 1:** The Journal of American Medical Association (JAMA) benchmark criteria.

Criteria	Description
Authorship	Authors and contributors, their affiliations, and relevant credentials should be provided.
Attribution	References and sources for all content should be listed clearly, and all relevant copyright information noted.
Currency	Website ownership should be prominently and fully disclosed, as should any sponsorship, advertising, underwriting, commercial funding arrangements or support, or potential conflicts of interest.
Disclosure	Dates that content was posted and updated should be indicated.

**Table 2:** Description of the Global Quality Score (GQS) 5-point scale used to evaluate videos with gallstone information on TikTok.

GQS	Description
1	Poor quality; poor flow of the site; most information missing; not at all useful for patients
2	Generally poor quality and poor flow; some information listed but many important topics missing; of very limited use to patients
3	Moderate quality; suboptimal flow; some important information is adequately discussed but others poorly discussed; somewhat useful for patients
4	Good quality and generally good flow; most of the relevant information is listed, but some topics not covered; useful for patients
5	Excellent quality and excellent flow; very useful for patients

### Statistical Analyses

Means and SDs were used for descriptive statistics. The Kruskal-Wallis test assessed differences between groups, and the Dunn multiple comparisons test for two-way intergroup comparisons of quantitative variables with no normal distribution. Spearman correlation analysis was used to evaluate the relationships between quantitative variables. *P*<.05 was considered statistically signiﬁcant. Statistical analyses were performed using GraphPad Prism version 9.0.0 for Windows (GraphPad Software).

## Results

### Features of Gallstone Videos

The initial 100 videos collected by the TikTok search had 520,805 likes, 17,947 comments, and 181,355 shares. The mean video duration was 49.43 (SD 24.63) seconds. Moreover, the average number of days after uploading (days since upload) was 152.0 (SD 146.4) days by the data collection date.

Table 3 shows the descriptive statistics for TikTok videos of different sources and content. According to video sources, 81% (81/100) of the videos were posted by doctors. The proportion of the rest of the sources were individuals 10% (10/100), news agencies 6% (6/100), and organizations 3% (3/100), respectively. In the content of videos, disease knowledge was the most dominant video content, which accounts for 56% (56/100) of all the videos. Moreover, the proportion of the rest content was 21% (21/100) for surgery technique, 16% (16/100) for lifestyle, and 7% (7/100) for news, respectively.

**Table 3:** Descriptive statistics for TikTok videos of different sources and content.

Variables		Likes, mean (SD)	Comments, mean (SD)	Shares, mean (SD)	Days since upload (days), mean (SD)	Duration (seconds), mean (SD)
**Video source (n=100)**						
	Doctor (n=81)	2247 (6006)	174.9 (358.8)	785 (2668)	133 (122.5)	52.73 (21.53)
	Individuals (n=10)	2520 (3477)	258.4 (341.7)	311.3 (453.7)	208.9 (216.9)	37 (35.16)
	News agencies (n=6)	52,232 (73,964)	182.5 (106.9)	19,070 (38,295)	349.3 (189.8)	28.17 (14.82)
	Organizations (n=3)	73.67 (79.32)	34.33 (40.46)	79 (47.51)	79.33 (66.11)	44.33 (52.54)
**Video content (n=100)**						
	Surgery technique (n=21)	657.7 (692)	79.71 (82)	68.95 (103.8)	131.8 (115.6)	58.24 (28.96)
	Disease knowledge (n=56)	1655 (3394)	177.3 (328.1)	543.8 (2203)	141.2 (134.6)	46.18 (19.6)
	Lifestyle (n=16)	5827 (11,657)	292.5 (565.4)	2204 (4168)	149.8 (166.5)	56.31 (32.09)
	News (n=7)	45,871 (69,610)	238 (204)	16,313 (35,711)	303.9 (211.1)	33.29 (17.76)

### Video Quality Assessments

The mean DISCERN, JAMA, and GQS scores of all 100 videos are 39.61 (SD 11.36), 2.00 (SD 0.40), and 2.76 (SD 0.95), respectively. We found that JAMA was unable to make a discriminating and accurate assessment of video quality; the majority of the videos (93/100, 93%) were rated as 2, and others were rated as 1 (3/100, 3%), 3 (1/100, 1%), and 4 (2/100, 2%), respectively. Therefore, JAMA was excluded in the subsequent video quality and correlation analysis.

**Figure 1:**
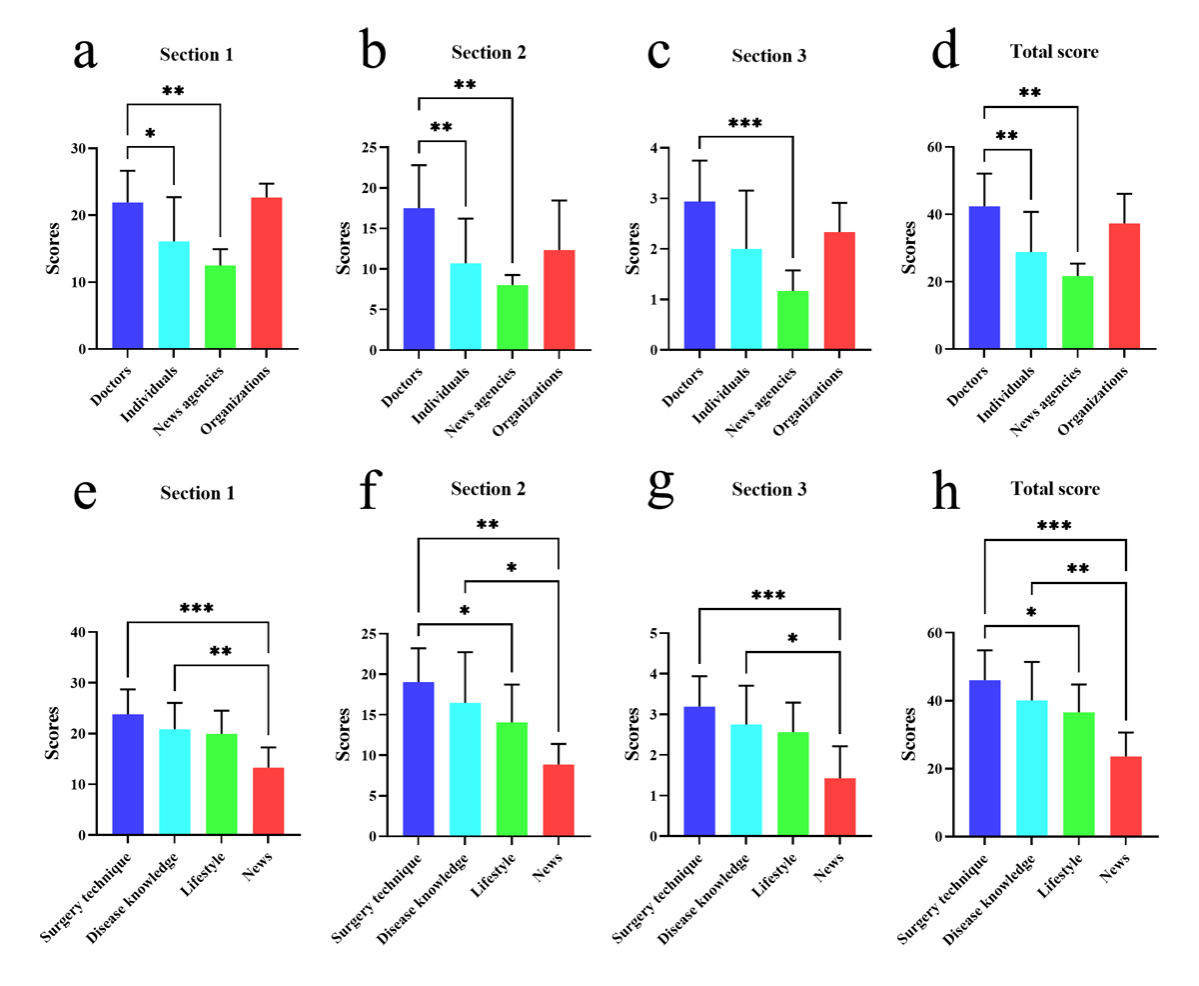
DISCERN scores for TikTok videos of different sources (a, b, c, d) and contents (e, f, g, h). *P<.05, **P<.01, ***P<.001.

We compared the video quality based on different sources and contents with DISCERN and GQS scores. Three sections and the total score of DISCERN were analyzed. In section 1, DISCERN score of doctors was significantly higher than those of individuals and news agencies (*P*=.04 and *P*=.002, respectively), and DISCERN scores for surgery technique and disease knowledge were significantly higher than those of the news (*P*<.001 and *P*=.01, respectively). Note that the reliability of gallbladder stone videos from professional sources and content is stronger than that of videos from nonprofessional sources and content. In section 2, DISCERN score of doctors was significantly higher than that of individuals and news agencies (*P*=.008 and *P*=.005, respectively), and DISCERN scores for surgery technique and disease knowledge were significantly higher than those of news agencies (*P*=.002 and *P*=.02, respectively). It indicates that the gallbladder stone videos from professional sources and content are better than those from nonprofessional sources and content in providing treatment information. In section 3, DISCERN score for doctors was significantly higher than that of the news agencies (*P*<.001), and DISCERN scores for surgery technique and disease knowledge were significantly higher than those of the news (*P*<.001 and *P*=.01, respectively). Overall, the total scores of doctors were significantly higher than those of individuals and news agencies (*P*=.008 and *P*=.001, respectively), scores for surgery techniques were significantly higher than those of lifestyle and news (*P*=.01 and *P*<.001, respectively), and scores of disease knowledge was significantly higher than those of news (*P*=.007). In general, the quality of gallbladder stone video information from professional content and professional sources is higher than that from nonprofessional sources and nonprofessional content (Figure 1).

**Figure 2:**
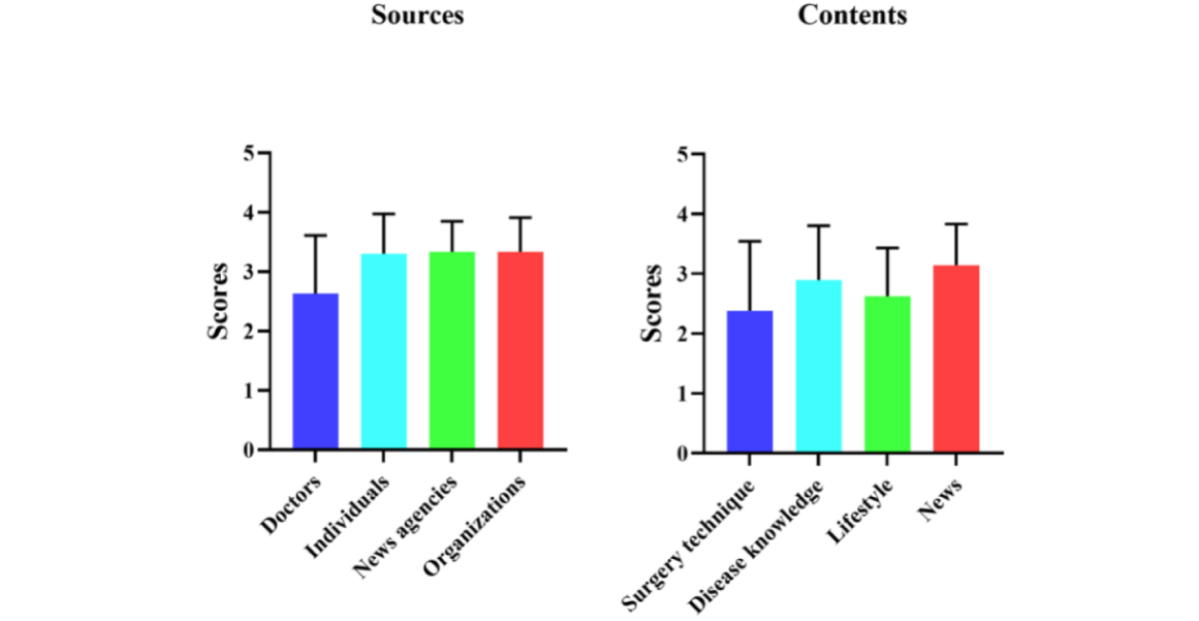
Global Quality Scores analysis for TikTok videos of different sources and content.

In GQS analysis, no significant differences were found between groups based on different sources or different contents (Figure 2).

**Figure 3:**
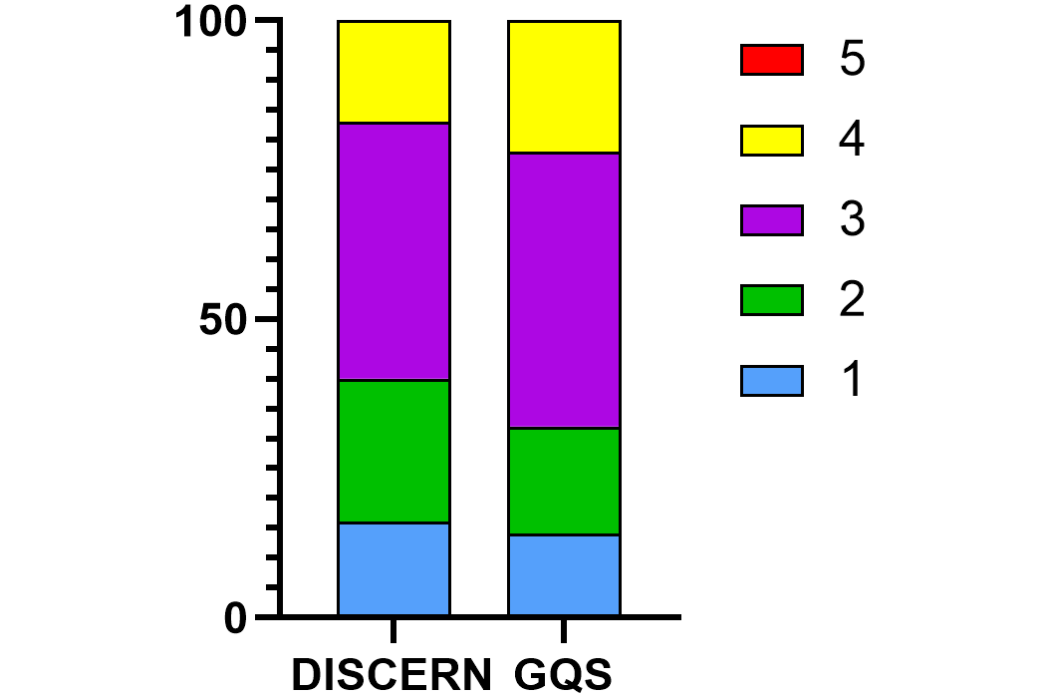
The 5 levels of DISCERN and Global Quality Scores (GQS).

**Figure 4:**
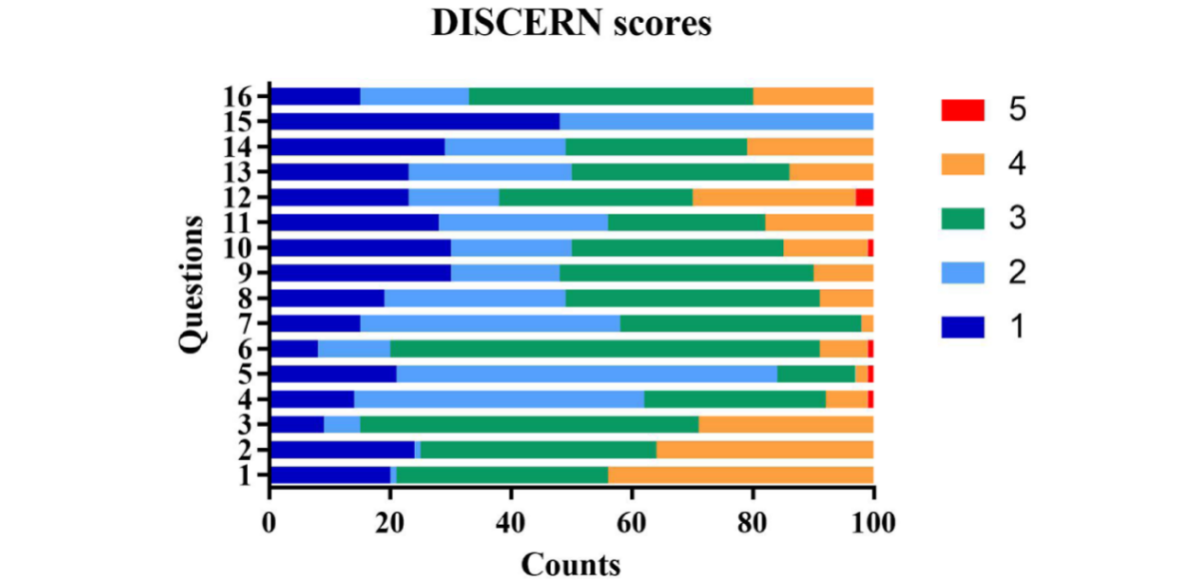
Detailed ratings of 100 gallstone videos by the DISCERN questionnaire with 16 questions.

According to DISCERN and GQS, gallstone-related videos’ quality score on TikTok is not high, mainly at fair (43/100, 43%) and moderate (46/100, 46%). We put these 5 levels in one-to-one correspondence, and the 5-level scores are reasonably consistent in DISCERN and GQS (Table 4, Figure 3). Further analysis of the DISCERN questionnaire revealed that the main reason for the low score was that the introductory statement was not supported by cited evidence-based sources (Question 4). Moreover, there was a lack of support for shared decision-making (Question 15; Figure 4).

**Table 4:** The 5-level scores of DISCERN and Global Quality Scores (GQS; n=100).

Scores		Value, n (%)
**DISCERN**		
	16-26 (very poor)	16 (16)
	27-38 (poor)	24 (24)
	39-50 (fair)	43 (43)
	51-62 (good)	17 (17)
	63-80 (excellent)	16 (16)
**GQS score**		
	1 (poor)	14 (14)
	2 (generally poor)	18 (18)
	3 (moderate)	46 (46)
	4 (good)	22 (22)
	5 (excellent)	0 (0)

### Correlation Analysis

Spearman correlation analysis revealed that the following variables were correlated positively: likes and comments (*r*=0.25, *P*=.01), likes and shares (*r*=0.74, *P*<.001), likes and days since upload (*r*=0.26, *P*=.009), comments and shares (*r*=0.24, *P*=.01), shares and days since upload (*r*=0.21, *P*=.04; Table 5).

DISCERN scores and video duration were positively correlated (*r*=0.59, *P*<.001). Negative correlations were found between DISCERN scores and likes and shares of videos (*r*=–0.30, *P*=.003 and *r*=–0.2329, *P*=.02, respectively; Table 6).

**Table 5:** The relationship level between video variables.

Variable and analysis^a^			Likes		Comments		Shares		Days since upload		Video duration
**Likes**											
	*r* value	1		—^b^		—		—		—	
	*P* value	—		—		—		—		—	
**Comments**											
	*r* value	0.26		1		—		—		—	
	*P* value	.01^c^		—		—		—		—	
**Shares**											
	*r* value	0.74		0.24		1		—		—	
	*P* value	<.001^d^		.01^c^		—		—		—	
**Days since upload**											
	*r* value	0.26		0.08		0.21		1		—	
	*P* value	.009^e^		.45		.04^c^		—		—	
**Video duration**											
	*r* value	–0.18		–0.06		–0.15		–0.02		1	
	*P* value	.07		.54		.13		.87		—	

^a^Pearson correlation analysis and *r* correlation coefficient.

^b^Not applicable.

^c^*P*<.05.

^d^*P*<.001.

^e^*P*<.01.

**Table 6:** Pearson correlation analysis between video quality scores and video variables.

Variable and analysis		DISCERN		GRS^a^
**Likes**				
	*r* value	–0.30	0.06	
	*P* value	.003^b^	.55	
**Comments**				
	*r* value	–0.15	0.12	
	*P* value	.14	.24	
**Shares**				
	*r* value	–0.23	–0.04	
	*P* value	.02^c^	.66	
**Days since upload**				
	*r* value	–0.06	0.06	
	*P* value	.55	.54	
**Video duration**				
	*r* value	0.59	–0.01	
	*P* value	<.001^d^	.91	

^a^GRS: Global Quality Scores.

^b^*P*<.01.

^c^*P*<.05.

^d^*P*<.001.

## Discussion

### Principal Findings

In this cross-sectional study, feature information about gallstone videos on TikTok was analyzed at one time point, and the quality of videos was evaluated using DISCERN and GQS instruments. The majority (81/100, 81%) of gallstone videos on TikTok were posted by doctors, ensuring the professionalism of the content. However, according to DISCERN and GQS video quality ratings, the doctors’ videos were not as good as expected. It is worth noting that TikTok does not authenticate the bloggers’ identities who claim to be doctors, so there may be some possibility that the identities are fraudulent, such as impersonating doctors to gain extra video views.

### Factors Influencing the Popularity of Videos

The number of likes, comments, and shares reflect, to some extent, the popularity of a video or post [25,26]. We found a positive correlation between likes, comments, and shares, indicating that popular videos were more likely to receive comments and be shared. The number of comments was also positively correlated with the number of shares, indicating that the videos receiving more comments are more likely to be shared. Days since upload positively correlates with likes and shares, indicating that videos are more likely to be favored over time. It is noteworthy that there was no correlation between video length and video popularity.

### The Overall Quality of the Video

The mean DISCERN, JAMA, and GQS scores of all 100 videos are 39.61 (SD 11.36), 2.00 (SD 0.40), and 2.76 (SD 0.95), respectively. Gallstone videos on TikTok were mainly evaluated at fair and poor grading ranges, consistent with previous studies about video quality on YouTube [27]. However, in a previous study about the quality of COPD videos on TikTok, the DISCERN scores of COPD videos are mostly around 56 (SD 11.8) to 66.8 (SD 3.8) [16], which are higher than the DISCERN scores of gallstone videos in our study. This result may be due to the following reasons: the DISCERN instrument does not have comparability between different diseases categories, and different scoring criteria of different researchers cause bias.

A positive correlation between video duration and video quality was found. The longer the video duration, the higher the video quality, which is the same conclusion as some previous studies [17]. Most TikTok videos are 15 seconds long, making it difficult to provide adequate information in such a short time. Although some videos’ duration can be extended, they are still much shorter than those on traditional video websites such as YouTube [28].

### Correlation Between Video Quality and Video Characteristics

We found a negative correlation between likes and DISCERN scores, as shown in Table 6. In our study, we were surprised to find that videos with more likes, shares, and comments had lower quality. These indicators of video popularity are negatively correlated with DISCERN scores, indicating that TikTok viewers cannot distinguish high-quality videos from low-quality ones. In other words, viewers seem to prefer lower-quality videos. This finding is consistent with previous research on the quality of videos on YouTube [17]. Educational videos can be less sensational and therefore less appealing to nonprofessionals. On the other hand, the recommendation mechanism of TikTok determines that videos with many likes are more likely to be recommended, so popular videos with low quality will become even more popular, further exacerbating the divergence between video quality and popularity.

In addition, the results may be related to the user characteristics of TikTok. As TikTok is a lifestyle entertainment app, its users prefer entertaining videos. Videos with beautiful graphics are more attractive. However, videos with high credibility are not popular. Because professional content tends to be unentertaining or even dull and therefore challenging to attract an audience, it is harder for them to gain popularity.

Other studies have found that viewers may be more likely to seek video content that deviates from conventional treatment regimens [29]. Nontraditional content that deviates from established medical advice may align with patients’ expectations and thus receive more views and likes.

### Evaluation of Quantitative Scoring Tools

We found that the JAMA score could not accurately assess the video information because there were only 4 indicators, and they were not precise enough. This result was consistent with previous reports [30]. Therefore, in the subsequent video quality analysis, we excluded JAMA. The consistency of DISCERN and GQS was acceptable. However, DISCERN and GQS can only assess the breadth of information contained in a video, but not its intrinsic quality. Assessment of intrinsic quality requires a manual audit by a professional.

### Possible Interventions

The likes and comments of individuals and news agencies are significantly higher than those of doctors and organizations, indicating that individuals and news agencies have more significant influence than doctors and organizations on TikTok, but the quality of videos is lower than that of professionals. On the one hand, news and individuals need to improve the quality of their information. On the other hand, doctors need to increase their influence. Medical video creators need to avoid boring content on the premise of professional content and better attract audiences to further spread the correct medical information.

Due to the complexity and professionalism of medical content, the length of medical-related videos should be extended accordingly to ensure sufficient information and content quality. TikTok should give professional certification to some video creators who publish medical professional content. A certification mark can be given, increasing the audience’s recognition of the information and helping the dissemination of professional information. At the same time, a professional review can improve the quality and reliability of medical professional videos. When searching and evaluating medical content on the internet, health searchers mostly choose the results that appear on the first page of the search engine and rarely view the results that appear outside the second search results page [31,32]. Therefore, TikTok can change the recommendation mechanism or display professionally reviewed content in the first results pages, which is more conducive to disseminating high-quality content.

In this study, no advertising content was found in the search results for gallstones. Since TikTok mainly features entertainment videos, medical videos are still in the process of increasing. The same goes for advertising videos.

### Strengths and Limitations

This study is the first to use multiple tools (DISCERN, GQS, and JAMA) to evaluate the quality and reliability of gallstone-related videos on TikTok. We further analyzed the correlation between video duration, likes, comments, and shares and video quality, and found that likes and shares were negatively correlated with video quality, and video duration was positively correlated with video quality. This study is also the first to analyze the quality of surgical disease-related videos on TikTok.

However, our study still has some limitations. Because these 3 evaluation tools were initially designed to evaluate textual content, there remain some limitations to the content of the video, such as not being able to evaluate the sophisticated production level and audio effect of the video. Furthermore, although we conducted a thorough analysis of the number of comments, we did not conduct a deeper analysis of the content of the comments. Sometimes, viewers leave comments not as a praise, but due to dissatisfaction. These negative reviews also increase the number of comments. Of course, we found in our research that the number of comments and likes, as well as the number of comments and shares, are positively correlated, so the number of comments can to some extent reflect the popularity of the video. Therefore, conducting a more in-depth study of positive and negative reviews in comments to explore their relationship with video quality is something we need to further analyze in future research. The current videos on TikTok are mainly for entertainment, and medical videos account for only a tiny part; therefore, further observation is needed to study the evolution process of medical videos on TikTok.

### Conclusion

Although videos on gallstones on TikTok are mainly provided by doctors and contain disease knowledge, they are of low quality. We found a positive correlation between video duration and video quality. High-quality videos received low attention, and popular videos were of low quality. Medical information on TikTok is currently not rigorous enough to guide patients to make accurate judgments. TikTok was not an appropriate source of knowledge to educate patients due to the low quality and reliability of the information.

## Appendix

Multimedia Appendix 1 The DISCERN instrument questionnaire.

## Abbreviations

COPD: chronic obstructive pulmonary disease
DISCERN: DISCERN instrument
GQS: Global Quality Scores
JAMA: Journal of American Medical Association
